# Influence of the Cement Film Thickness on the Push-Out Bond Strength of Glass Fiber Posts Cemented in Human Root Canals

**DOI:** 10.1155/2016/9319534

**Published:** 2016-04-10

**Authors:** Natália Araújo Silva Prado, Reinaldo de Souza Ferreira, Marcos Henrique de Pinho Maurício, Sidnei Paciornik, Mauro Sayão de Miranda

**Affiliations:** ^1^Department of Dentistry, Rio de Janeiro School of Dentistry, State University of Rio de Janeiro, 222600-000 Rio de Janeiro, RJ, Brazil; ^2^Department of Materials Engineering, Pontifical Catholic University of Rio de Janeiro, 22451-045 Rio de Janeiro, RJ, Brazil

## Abstract

The present study evaluated the influence of the cement film thickness on the push-out bond strength of glass fiber posts in the cervical, medium, and apical thirds of root canal spaces. Thirty roots were randomly divided into three groups, according to the fiber post system's drills: (G1) #2; (G2) #3; (G3) #4. The posts were cemented using a self-adhesive cement, and a small amount of powdered Rhodamine B was used as a stain. Images of both sides of each slice were obtained before and after the push-out test. To determine the cement thickness, a macro routine was developed using the software KS 400. The data were analyzed statistically using Kruskal-Wallis and Dunn's test. G2 (14.62 ± 5.15 MPa) showed statistically higher bond strength values than G1 (10.04 ± 5.13 MPa) and G3 (7.68 ± 6.14 MPa). All groups presented higher bond strength values in the apical third. The bur diameter significantly influenced the results of the shear bond strength for the push-out test. The slight increase in the cement thickness allowed an increase in the values of shear bond strength when compared to very thin or very thick cement films.

## 1. Introduction

Fiber reinforced posts are widely used to restore endodontically treated teeth [1]. They are usually luted with resin cements, which promote an increase in retention when compared to conventional cements, such as zinc phosphate [2, 3]. However, this technique is very sensitive and the manufacturers protocol must be carefully followed in order to achieve successful restorations [4]. To simplify the cementation technique, self-adhesive resin cements were developed. These cements are dual-curing, with the characteristic of self-adhesion to the tooth structure, which eliminates the need of adhesive systems [5]. Studies have been developed to evaluate the influence of adhesive systems, resin cements, and curing modes on the retention of fiber posts [6–8]. However, there have been few studies on the cement film thickness and its influence on the bond strength of these posts.

The use of prefabricated posts with a diameter that does not correspond to the size of the root canal preparation is common in dental practice. In some clinical situations, such as the presence of caries in the cervical third of a root canal, oval shaped roots, or canals that are larger in the cervical portion, an enlargement of the post space diameter is necessary to obtain a precise adaptation of the post to the canal, leading to excessive removal of tooth structure [4]. However, it has been speculated that the intimate contact produced by the drill and the fiber post may not provide enough space for the resin cement to provide its maximum strength [9]. Therefore, the possibility of increasing the film thickness of the cement in order to increase the bond strength between resin cement and root dentin has been questioned.

The purpose of this* in vitro* study was to evaluate the influence of the cement film thickness on the bond strength of glass fiber posts in different portions of the root canal (cervical, medium, and apical), cemented with a self-adhesive resin cement, using a push-out test. The hypotheses tested were as follows: there was no difference between (1) the bond strength values and the three cement film thicknesses and (2) the bond strength values at the cervical, middle, and apical thirds of the root canals.

## 2. Materials and Methods

### 2.1. Specimen Preparation

Thirty single rooted human teeth, with a root length greater than 15 mm and without root curvature, were selected for this present study. They were stored after extraction in distilled water at 37°C and used within six months. The teeth were transversally sectioned at the cementoenamel junction using a diamond blade (Diamond Wafering Blade 15 HC/BUEHLER, Lake Bluff, IL, USA) in a cutting machine (Labcut 1010/Extec Corp., Enfield, CT, USA). The roots were embedded in epoxy resin using plastic cylinders with a 17 mm diameter. All roots were instrumented using the step-back technique and obturated using the lateral condensation technique with gutta-percha and the endodontic cement, Sealer 26 (Dentsply Maillefer). The filled roots were stored in distilled water at 37°C for one week.

After storage, the canals were enlarged with #2, #3, and #4 Gates-Glidden burs, leaving 5 mm of gutta-percha at the apex. The roots were randomly divided into three groups (*n* = 10), according to the WhitePost DC system's drills (FGM, Joinville, SC, Brazil): (G1) preparation of the canal with the #2 drill; (G2) #3 drill; (G3) #4 drill. The preparation was standardized at a depth of 10 mm.

Cementation was performed using the dual-curing self-adhesive resin cement, RelyX*™* U100 in a Clicker*™* Dispenser (3M ESPE, St. Paul, MN, USA), following the manufactures' instructions. A small amount of powdered Rhodamine B was incorporated into the cement during the mixing procedure to give the cement a pink color and allowing it to be easily distinguished from the fiber post and root canal dentin. The amount of Rhodamine B used was measured using the tip of a short needle to catch a few grains for one measure dosed by the Clicker (197 mg). All groups were cemented with the double-tapered translucent glass fiber post of the #2 WhitePost DC system. The thickest end of the post has a diameter of 1.8 mm, while the thinnest end of the post has a diameter of 1.05 mm. The posts were cleaned with 92.8% ethanol and silanized with RelyX*™* Ceramic Primer (3M ESPE) before cementation. The cementation procedures were performed following the RelyX*™* U100 manufactures' instructions. The cement was applied onto the post surface and injected into the root canal using a specific syringe with a tube and needle (Centrix system, DFL, Rio de Janeiro, RJ, Brazil). The post was placed into the root canal with light finger pressure. Light activation was performed at the top of the fiber post for 40 seconds with an irradiance of 950 mW/cm^2^, as measured by a radiometer (Hilux Ledmax Curing Light Meter, SDI, Bayswater, VIC, Australia). The resin cement was covered with a glass ionomer cement (Ketac Fil Plus, 3M ESPE). The roots were stored in distilled water at 37°C for one week.

### 2.2. Push-Out Bond Strength Test

After storage, the roots were transversally sectioned with a diamond blade (Diamond Wafering Blade 15 HC) in a cutting machine (Labcut 1010) at a speed of 150 rotations per minute (rpm). Six slices, with 1 ± 0.10 mm thickness, were obtained, corresponding to the cervical, middle, and apical thirds, for a total of 2 slices per region.

The push-out bond strength test was performed with the universal testing machine, EMIC DL-200 MF (EMIC/São José dos Pinhais, PR, Brazil), using a cell load of 50 kg at a crosshead speed of 0.5 mm/min until post dislodgment. The slices were positioned on a push-out jig immediately after the cutting procedures. Due to the conical shape of the posts, the load was applied in an apical to cervical direction. The metallic end of the plunger, which measured 0.8 mm in diameter, was positioned to touch only the post, so as not to create tension in the surrounding dentin walls. After the push-out test, the specimens were individually stored in a dry setting.

The retentive strength of the post segment was expressed in MPa. These values were obtained dividing the force to cause the failure in N for the adhesion area in mm^2^. Considering its conical shape, the exact area of the post fragment was previously calculated using the following mathematical formula: *π*(*R* + *r*)[*h* ^2^ + (*R* − *r*)^2^]0.5, where *R* = radius of the cervical side of the post (mm); *r* = radius of the apical side of the post (mm); *h* = thickness of the slice (mm).

The *R* and *r* dimensions were measured using images of the cervical and apical sides of the slices, captured before the mechanical test by the stereomicroscope, SteREO Discovery V8, with 1.6x magnification and the integrated camera, AxioCam ICc1 (Carl Zeiss Micro Imaging GmbH/Jena, Germany). The images were treated by the software AxioVision 4.8 (Carl Zeiss). The “scalings control” tool of this software allowed the ability to obtain each pixel dimension in *μ*m, so the radii of the posts were obtained. The relation obtained was 1 pixel: 4.587 *μ*m. The thickness of the slices (*h*) was measured with the Digital External Micrometer (DIGIMESS/São Paulo, SP, Brazil).

### 2.3. Cement Film Thickness Measurements

**Figure 1:**
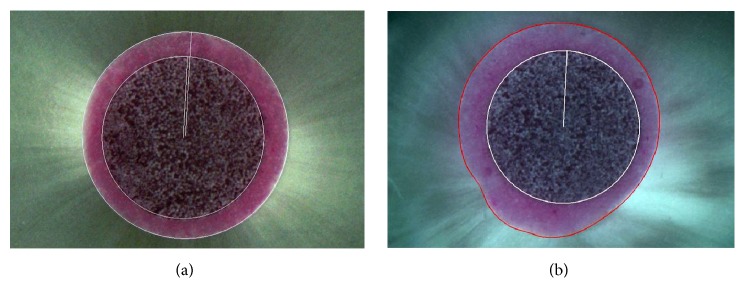
(a) “Circle points” tool used to manually contour the perimeter of the perfectly round post or canals. (b) “Outline spline” tool used to contour the perimeter of oval or irregular canals.

**Figure 2:**
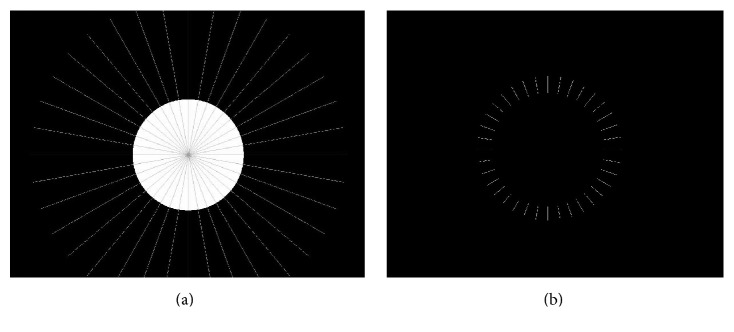
(a) 36 radius lines were traced from the center of the post, intercepting the perimeter of the post and the root canal. (b) Intersection of the radius lines with the resin cement area.

In order to obtain the cement film thickness, the images captured prior to the push-out test were again evaluated using the software, AxioVision 4.8. The “circle points” tool was used to manually contour the perimeter of the perfectly round post or canals (Figure 1(a)), and the “outline spline” tool was used to contour the perimeter of oval or irregular canals (Figure 1(b)). The delimited contours of each image were converted into a new binary image with a white outline and black background, to be processed in the software, KS 400 8.0 (Carl Zeiss). A macro routine (sequence of commands from the software) was developed to digitally process [10] the binary images, to measure the cement film thickness of each specimen. In each image, 36 radius lines were traced from the center of the post, with 10-degree intervals, intercepting the perimeter of the post and the root canal (Figure 2(a)), obtaining the intersection of the radius lines with the cement area (Figure 2(b)). The average of the 72 measurements (cervical and apical) was obtained.

### 2.4. Failure Mode Analysis

**Figure 3:**
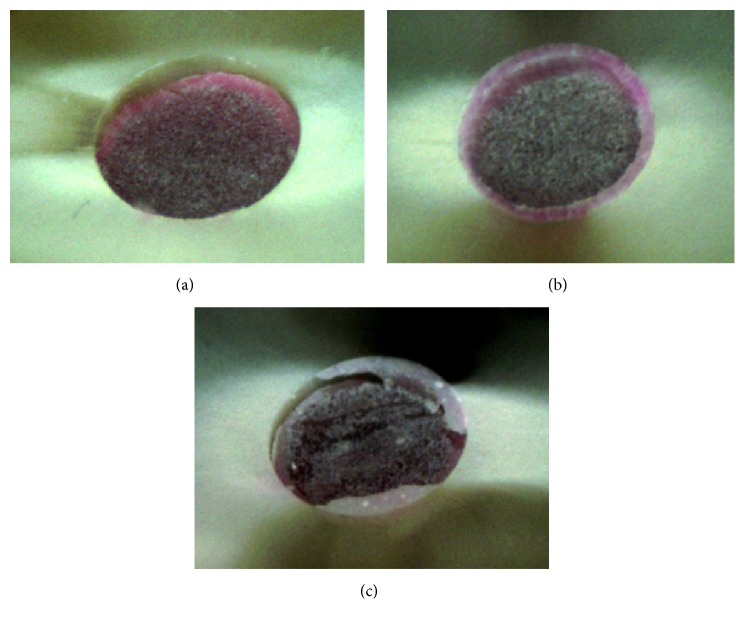
(a) Adhesive fracture between resin cement and dentin. (b) Adhesive fracture between resin cement and fiber post. (c) Mixed fracture.

To determine the failure mode, new perpendicular and oblique images were captured after the mechanical testing from both sides of each slice using the stereomicroscope. Fracture modes were classified as adhesive between resin cement and dentin (Figure 3(a)); adhesive between resin cement and fiber post (Figure 3(b)); and mixed fractures (Figure 3(c)).

### 2.5. Statistical Analysis

Kolmogorov-Smirnov and Levene test were used. The data were then analyzed statistically using Kruskal-Wallis and Dunn's test. Nonparametric options to ANOVA and* post hoc* multiple comparison tests were chosen due to departing from normality and heteroscedasticity in the data. IBM SPSS version 15.0 and R software version 3.2.3 were used.

## 3. Results

Tables 1 and 2 show three analyses each: more general differences among groups (horizontal comparison in the bottommost row of the table); differences among groups for each third (horizontal comparison); and differences among thirds inside each group (vertical comparison).

There were statistically significant differences in the cement film thickness among all groups (*p* < 0.05). G3 (248.78 *μ*m) presented statistically higher values of cement film thickness, followed by G2 (185.92 *μ*m) and G1 (110.16 *μ*m) (Table 1).

It was also observed that the push-out bond strength was statistically influenced by the drill size of the WhitePost (WP) system used on the preparation of the post space (*p* < 0.05). There were statistically significant differences among all groups (Table 2). G2 presented the highest bond strength values (14.62 ± 5.15 MPa), followed by G1 (10.04 ± 5.13 MPa) and G3 (7.68 ± 6.14 MPa).

When comparing thirds, G2 showed statistically significant differences. In the apical third (18.37 ± 4.13 MPa) the values were higher than the middle third (15.10 ± 3.60 MPa). Lower values were found in the cervical third (10.34 ± 4.43 MPa) for this group. For G1 higher values were found in the apical third (12.72 ± 4.58 MPa). The cervical (7.67 ± 4.92 MPa) and middle (9.50 ± 4.83) thirds presented statistically similar values. G3 also presented higher values in the apical third (11.25 ± 6.12 MPa), and the cervical (6.06 ± 6.01 MPa) and middle (5.92 ± 5.00 MPa) thirds presented statistically similar values (Table 2).

The failure mode was different among the groups. G1 presented predominantly mixed failures (46%), while G2 presented adhesive failures between the post and cement (48%), and G3 presented adhesive failures between cement and dentin (71%) (Table 3).

## 4. Discussion

The mechanical test used in the present study was the push-out test, which allows for the evaluation of the bond strengths in different regions of the root canal, with the results of the shear stress between the dentin/cement and cement/post being comparable to clinical conditions [4, 11, 12]. Despite being a sensitive technique, the high standard deviation found in this study can be explained mainly due to the inherent characteristics of the root dentin, as the morphology, density, and orientation of the dentinal tubules, predominantly in the apical region [13, 14]; the root canal morphology and high *C*-factor [15]; presence of a smear layer [14]; difficult access of instruments and light, and presence of remnants of gutta-percha and endodontic cement [16]. A factor that could be avoided in the current study was the use of an endodontic cement without eugenol, Sealer 26 (Dentsply Maillefer). Some authors affirm that Sealer 26 does not interfere with the bond strength of fiber posts cemented with self-adhesive cements [17].

The post used in this present study was a double-tapered glass fiber post that has an elastic modulus similar to the dentin, which may result in a more favorable fracture mode [16, 18, 19]. Additionally, it has a high fiber concentration per surface area, which may increase the fracture resistance of the posts [20].

In order to cement the posts, the resin cement RelyX*™* U100 (3M ESPE) was used in this study. As this cement is autoadhesive, acid etching prior to its application would be detrimental for the dentin bonding effectiveness, since it could promote an inadequate infiltration of the demineralized collagen mesh [21]. So, the pretreatment of the root dentin was performed exclusively with NaOCl, following the manufacturer's instructions (3M ESPE).

The resin cement used is a hand mixed resin cement. This technique favors the incorporation of air into the cement mix. In order to reduce the presence of air bubbles within the cement, a Centrix syringe with a needle tip was used during the cementation procedure [22], and the absence of these voids in the majority of the specimens could be observed on the stereomicroscope images before mechanical testing. Some authors suggest that the presence of air bubbles in the structure of the resin cement may reduce the stress provided by the high *C*-factor. However, the mechanical properties of the cement could be prejudiced, increasing the chances of adhesive failure [16].

Regarding the methodology used to measure the cement film thickness, some methods can be found in the literature to measure the cement film thickness, as follows: average values obtained in 4 manual measurements using digitally processed images [23], values obtained with digitally processed images by subtracting the area of the post and root canal [24], and values obtained by subtracting the diameters of the post and bur [4, 12, 16]. In the current study, the analysis of the cement film thickness was made with 36 measurements on each side of the slice, with a total of 72 measurements, using a macro routine developed in the KS 400 software, which provided the ability to obtain a more precise average value, thereby simplifying the gathering of results by automation while standardizing the procedures.

The bond strength values among the groups were statistically different, with the lowest values found in G3 (7.68 ± 6.14 MPa), which had the greatest cement film thickness (248.78 *μ*m). G1 (10.04 ± 5.13 MPa), with the thinnest cement film thickness (110.16 *μ*m), presented higher values than G3; however, those values were lower than G2 (14.62 ± 5.15 MPa), which had an intermediate cement thickness (185.92 *μ*m). With these results, the first null hypothesis can be rejected, as there were differences among the groups. Some authors speculated that the intimate contact produced by drills and fiber posts systems with the same diameter may not provide enough space for the resin cement to develop its maximum strength [9]. This could explain the results obtained in the current study. Additionally, it is known that the *C*-factor increases in root canals where there is a considerable thin cement film thickness [25].

Some authors presented similar results, observing that the use of burs with a diameter slightly thicker than the one indicated by the manufacturer results in higher pull-out bond strength of fiber posts cemented in root canals with resin cement [9, 12]. Despite the similar results, the materials used in these studies were different from the ones used in this present study. Hagge et al. [9] used cylindrical metallic posts (ParaPost) and autopolymerizing resin cement (Panavia 21 OP). D'Arcangelo et al. [12] used quartz fiber posts (Endo Light-Post) and autopolymerizing resin cement (Panavia 21 OP).

Other studies did not observe significant differences in push-out bond strengths among the various cement film thickness tested [16, 23]. The materials used were also different in those studies. Perdigão et al. [16] used the autopolymerizing resin cement, Post Cement Hi-X, and Perez et al. [23] used the dual resin cement, Duolink (Bisco). Both studies used quartz fiber posts (DT Light-Post).

One study showed that precisely fitted tapered glass fiber posts (DentinPost ER) cemented with various cements, including the self-adhesive cement, RelyX Unicem, presented higher bond strengths [4]. Despite the similarity between the materials used in this present study, those specimens were subjected to thermocycling and the pull-out test, influencing the results.

In the current study, the root canal section significantly influenced the bond strength, with higher values found in the apical third in all groups (Table 2). Therefore, the second null hypothesis was also rejected, as there were differences among the thirds. Bitter et al. [11] presented similar results, where the apical and middle thirds showed greater bond strength values. However, Cecchin et al. [17] did not observe a difference in the bond strength values among the thirds. Other studies found different results, with a reduction on the bond strength values of the self-adhesive resin cements RelyX U100 or Unicem toward the apical third [15, 26].

Some authors claim that the increase in bond strength is directly related to the increase of the density of the dentinal tubules [15, 26]. A reduction of the bond strength toward the apical third would be expected, considering that the density of the dentinal tubules is lower in this third when compared to the middle and cervical thirds [27]. However, this was not observed in the current study, indicating that the bond strengths between fiber posts cemented with self-adhesive cements may be more related to the presence of solid dentin [27, 28].

The failure mode was different among the groups. G1 presented predominantly mixed failures, while G2 presented adhesive failures between the post and cement, and G3 presented adhesive failures between cement and dentin. Some studies that used RelyX U100 or Unicem showed more failures between cement and dentin [11, 29, 30]. Other studies identified more mixed failures [4, 17]. One study found more failures between the cement and post [31]. It was not possible to draw a conclusion about the cause of the failures, since they were too diverse, which can indicate that the Rhodamine B could have had an influence on the failure mode. However, it has been demonstrated that the Rhodamine B does not influence the bond strengths when used in low concentrations [32, 33], as used in the present study.

Further studies are needed to evaluate thermal and mechanical aging while using more accurate methods to measure the cement film thickness. Despite the important role of Rhodamine B in the methodology of this study, the use of Rhodamine B as a marker in adhesive cements should be performed with caution. In addition, bur diameter should be considered for minimal removal of dentin structure to obtain sufficient bond strength of fiber posts cemented with self-adhesive resin cements. Regarding the results of this present study, it can be advised to prepare post spaces with a diameter slightly greater than the glass fiber post.

## 5. Conclusions

When considering the experimental conditions of the present study (*in vitro*), it can be concluded that

1. the three bur diameters tested significantly influenced the bond strength results. Higher values were obtained when using the #3 bur associated with the #2 fiber glass post from the WhitePost DC system, indicating that the slight increase in the cement thickness allowed an increase in the values of shear bond strength in comparison with very thin (#2 bur) or very thick (#4 bur) cement films;
2. the bond strength values were higher in the apical third of the root canal, when compared with the middle and cervical thirds.

## Figures and Tables

**Table 1:** Cement film thickness by drill size and location of root section (*µ*m).

Root section	G1 (WP #2)	G2 (WP #3)	G3 (WP #4)
	Mean (±SD)	Mean (±SD)	Mean (±SD)
Cervical	136.29 (±73.48)^aC^	191.67 (±58.46)^aB^	267.46 (±38.56)^aA^
Middle	105.10 (±48.06)^aC^	171.28 (±43.43)^aB^	239.88 (±27.55)^bA^
Apical	92.09 (±30.82)^aC^	196.42 (±34.95)^aB^	238.45 (±28.95)^bA^
Total	110.16 (±54.89)^C^	185.92 (±47.01)^B^	248.78 (±34.31)^A^

Note: different superscript lowercase letters indicate statistically significant differences by column. Different superscript capital letters indicate statistically significant differences by row (Kruskal-Wallis and Dunn's test, *p* < 0.05).

**Table 2:** Push-out bond strengths by drill size and location of root section (MPa).

Root section	G1 (WP #2)	G2 (WP #3)	G3 (WP #4)
	Mean (±SD)	Mean (±SD)	Mean (±SD)
Cervical	7.67 (±4.92)^bAB^	10.34 (±4.43)^cA^	6.06 (±6.01)^bB^
Middle	9.50 (±4.83)^bB^	15.10 (±3.60)^bA^	5.92 (±5.00)^bC^
Apical	12.72 (±4.58)^aB^	18.37 (±4.13)^aA^	11.25 (±6.12)^aB^
Total	10.04 (±5.13)^B^	14.62 (±5.15)^A^	7.68 (±6.14)^C^

Note: different superscript lowercase letters indicate statistically significant differences by column. Different superscript capital letters indicate statistically significant differences by row (Kruskal-Wallis and Dunn's test, *p* < 0.05).

**Table 3:** Failure modes observed on the debonded specimens of the three experimental groups (%).

Failure modes	G1 (WP #2)	G2 (WP #3)	G3 (WP #4)
Adhesive of dentin/resin cement	43	34	71
Adhesive of resin cement/fiber post	11	48	09
Mixed	46	18	20
